# Mapping the Evolution of Social and Emotional Learning Research in Primary Education Contexts: A Bibliometric and Thematic Analysis

**DOI:** 10.3390/jintelligence13090123

**Published:** 2025-09-19

**Authors:** Melek Alemdar

**Affiliations:** 1 Ahmet Keleşoğlu Education Faculty, Necmettin Erbakan University, 42090 Meram, Konya, Türkiye; melek.alemdar@erbakan.edu.tr or melek.alemdar@manchester.ac.uk; 2 Manchester Institute of Education, The University of Manchester, Manchester M13 9PL, UK

**Keywords:** social and emotional learning (SEL), bibliometric analysis, thematic mapping, research trends, primary schools

## Abstract

This study presents a comprehensive bibliometric and thematic analysis of social and emotional learning (SEL) research in primary education, aiming to map its evolution, key contributors, and conceptual structure. Drawing on 915 peer-reviewed articles published between 1983 and 2025, retrieved from Web of Science and Scopus, the analysis employed performance metrics, science mapping, and thematic clustering techniques. Findings reveal a marked acceleration in SEL publications since the mid-2010s, with the United States, United Kingdom, and Australia leading both in research output and collaborative networks. Science mapping identified concentrated author and institutional clusters, while also highlighting geographic disparities in global research participation. Thematic analysis uncovered a shift from early focuses on behavioral and emotional regulation toward systemic, school-based interventions emphasizing mental health, resilience, professional development and family engagement. Clustering results positioned ‘social-emotional learning’ as the densest yet fragmented basic theme, reflecting its structural centrality alongside persistent conceptual dispersion across intervention models, implementation processes, and target populations. This study’s findings offer a macro-level synthesis of the SEL research landscape in primary education with the related implications being discussed.

## 1. Introduction

Centuries ago, Aristotle asserted that “the aim of education must be to help people become fully human, otherwise it is pointless” (Nemec and Roffey 2005, p. 2). In this view, becoming fully human entails self-understanding and embracing emotions as a source of personal empowerment. Historically, learners have gained knowledge not only through cognitive engagement but also through emotionally charged experiences that shape their self-concept and identity (Alemdar and Anılan 2022). This developmental process involves forming a strong sense of individuality and recognizing personal needs through interpersonal interactions—an inherently dynamic journey marked by fluctuations and complexity. In particular, middle childhood (ages 5–11) is a period of intense biological, cognitive, social, and emotional growth (Eccles 1999). As children begin to encounter increasingly complex social environments, they require a broad array of skills to navigate these challenges effectively (Jones et al. 2017). Supporting this holistic development means nurturing not only academic and cognitive capacities but also social and emotional competencies. A growing body of research emphasizes the role of social and emotional learning (SEL) in bridging thinking, feeling, and acting within specific social contexts (Elias et al. 1997; Lopes and Salovey 2004).

SEL, grounded in emotional intelligence theory (Salovey and Mayer 1990; Goleman 1995) and frameworks such as the Collaborative for Academic, Social, and Emotional Learning (CASEL), refers to the process of cultivating skills in self-awareness, emotion regulation, empathy, relationship-building, and goal setting (CASEL 2025a). Defined as an integral part of education and human development, SEL is the process through which all young people and adults acquire and apply the knowledge, skills, and attitudes to develop healthy identities, manage emotions and achieve personal and collective goals, feel and show empathy for others, establish and maintain supportive relationships, and make responsible and caring decisions (CASEL 2025a). These competencies are foundational for students to navigate social complexities, enhance academic engagement, and foster long-term well-being (Downes and Cefai 2016; Weissberg et al. 2015). When embedded within primary education in emotionally supportive learning environments, SEL contributes meaningfully to children’s holistic development (Cefai et al. 2022).

The potential benefits of SEL are strongly supported by a significant body of research, encompassing meta-analyses (e.g., Cipriano et al. 2023), systematic reviews (e.g., Cefai et al. 2018) and umbrella reviews (e.g., Durlak et al. 2022; Moreno et al. 2024; Wigelsworth et al. 2022). These studies synthesize diverse data to demonstrate the impact of SEL on various student outcomes, often focusing on school-based SEL interventions and intervention mechanisms (Domitrovich et al. 2017). While meta-analyses offer robust effect size estimations (Walker et al. 2008) and systematic reviews provide rigorous, unbiased syntheses of existing evidence (Siddaway et al. 2019), both primarily offer micro-level analyses of individual studies and specific hypotheses (Cooper 2015). This micro-focus, while valuable for in-depth understanding of specific interventions, may lead to overgeneralization and neglect broader trends within SEL research (Wigelsworth et al. 2022). Therefore, a complementary macro-level perspective is needed. Bibliometric analysis fulfills this need by providing a comprehensive, quantitative overview of the evolution of the field, highlighting publication trends, key research contributors, emerging themes and knowledge gaps (Ellegaard and Wallin 2015), complementing the detailed insights gained through systematic reviews and meta-analyses, and ultimately guiding future investigations.

### The Case for a Bibliometric Anaysis

The exponential growth in SEL research (Wigelsworth et al. 2022) necessitates a robust, comprehensive approach to understanding its evolution. Bibliometric analysis provides a crucial macro-level perspective, quantitatively analyzing publication patterns to reveal the field’s trajectory and inform strategic planning (Verma and Gustafsson 2020). This method objectively evaluates research structures, mapping key areas, stimulating new ideas, and identifying knowledge gaps (Ellegaard and Wallin 2015; van Raan 1996). By analyzing publication trends and researcher contributions, bibliometrics reveals patterns and future research directions not readily apparent through more focused reviews (Leydesdorff et al. 2016). It also provides a comprehensive view of the core concepts within the field and how they are interconnected. Its ability to synthesize large, objective datasets (e.g., citation counts, keyword frequencies) while incorporating both quantitative and qualitative interpretations (Donthu et al. 2021) makes bibliometrics suited to charting the development of the SEL field. Ultimately, this approach helps scholars gain a holistic overview, identify research gaps, generate innovative ideas, and position their contributions.

While several studies have employed bibliometric analysis within SEL research, this study offers a more comprehensive and nuanced approach. Existing research, such as Haruna and Isa (2024) and Ağırkan and Ergene (2021), has focused on limited timeframes (e.g., 2013–2022), specific contexts (e.g., SEL within English language classrooms) or limited data sources (e.g., Web of Science alone). Manjarres et al. (2023) concentrated solely on emotional skills with a narrow keyword scope. The current study distinguishes itself by combining bibliometric and thematic analysis, incorporating data from both Scopus and Web of Science, examining studies up to February 2025, and utilizing broader keyword parameters. This comprehensive approach provides a more in-depth understanding of global trends and emerging themes in SEL research, addressing gaps in previous studies and expanding the scope of analysis.

In accordance with the objectives, three research questions are introduced:

RQ1. How has research on SEL in primary education developed over time in terms of publication output and the contributions of authors, journals, institutions, and countries?

RQ2. What does the science mapping of SEL literature in primary education contexts reveal about the intellectual, and social structure of the field?

RQ3. What are the most frequently used keywords and thematic clusters in SEL research within primary education, and how do these elements reflect the conceptual structure and thematic evolution of the field over time?

## 2. Materials and Methods

### 2.1. Research Design

This study employed a three-phase bibliometric analysis to evaluate the productivity, scholarly impact, and collaborative networks of publications, authors, institutions, and countries in primary-level SEL research, while also identifying emerging trends and informing future research directions. This three-phase design follows recommended best-practice guidelines for bibliometric research (Donthu et al. 2021), ensuring clarity in aims, techniques, and scope.

In the first phase, *performance analysis* was used to examine publication trends, including annual output, cumulative growth, and the expansion of research activity over time (Ellegaard and Wallin 2015). Contributions of influential authors, journals, institutions and countries were assessed by analyzing publication volume, citation counts, and impact metrics, enabling the identification of key contributors to the field (Garfield 2006; Hirsch 2005). The second phase employed *science mapping* to uncover the intellectual and social structure of the literature. By analyzing similarities between items—such as authors, keywords, and journals—and visualizing their relational networks, this phase illustrated patterns of collaboration linkages across the field (Boyack and Klavans 2014; Ozturk 2021). In the third phase, *thematic analysis* was conducted to detect dominant clusters and project future directions of research. Techniques such as network analysis, and clustering were applied to map the conceptual architecture and trend dynamics of the field. Keyword frequencies and visualizations of data clusters were used to identify evolving themes and highlight areas of growing scholarly attention (Zupic and Čater 2015; Li et al. 2023).

Table 1 presents a consolidated view of the analytical procedures (RQ1–RQ3), showing how performance, science mapping, and thematic analyses were conducted and what outputs each stage produced.

### 2.2. The Procedure

Bibliometric mapping requires structured and standardized metadata (titles, abstracts, author keywords, citation and reference data) that are consistently available only in databases such as Web of Science (WoS) and Scopus. These databases are also the most widely used and methodologically robust sources for bibliometric research (Donthu et al. 2021; Moral-Muñoz et al. 2020), ensuring transparency, comparability, and reproducibility. For this reason, the present study relies on WoS and Scopus rather than broader or less standardized sources. Although some scholars suggest that single-database designs may reduce technical workload and the risk of duplication errors (Donthu et al. 2021), I combined WoS and Scopus to maximize coverage. To integrate and deduplicate records, data from WoS and Scopus results were merged and processed using the *R* programming language and the “*bibliometrix*” package (Aria and Cuccurullo 2017), ensuring consistent formatting for subsequent bibliometric analyses.

On 22 February 2025, a systematic search using the WoS and Scopus databases was performed using the topic fields with the following keyword string:

“Social and emotional learning” OR “Social-emotional learning” OR “Socio-emotional learning” AND “Primary school” OR “Elementary school” AND “Children” OR “Child” OR “Pupil” AND Document type: “Article”

This initial query yielded 1323 documents. To ensure relevance and consistency, the following six inclusion criteria were applied:

1. Explicit SEL focus: The publication must explicitly focus on social and emotional learning (SEL) or close variants.
2. Educational context: The study must pertain specifically to primary education contexts (typically ages 4–12).
3. Publication type: Only peer-reviewed journal articles and systematic reviews were included, since bibliometric analysis relies heavily on structured metadata (e.g., abstracts, author keywords, citation information) consistently available in these formats.
4. Peer review requirement: All included documents must have undergone peer review.
5. Timeframe: Publications up to February 2025 were included.
6. Language and availability: Articles had to be written in English and provide full-text availability.

**Figure 1:**
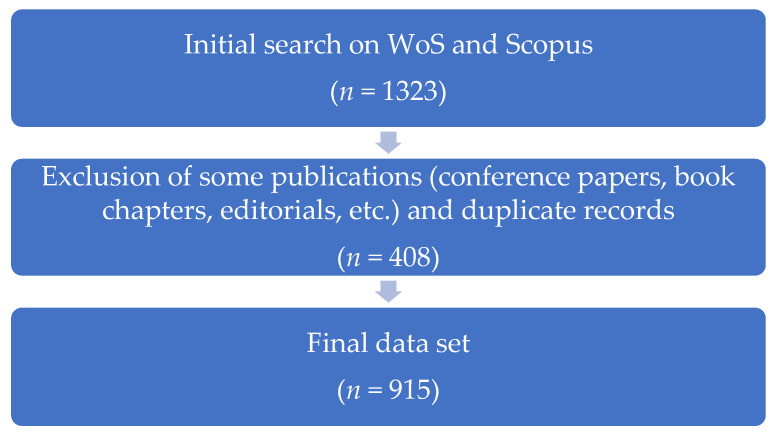
Data collection and formation process.

Gray literature sources (e.g., book chapters, theses, and editorials) were excluded because they are inconsistently indexed in major databases, often lack abstracts or standardized keywords, and vary in peer review standards, making them less suitable for bibliometric mapping. In addition, duplicate records across databases and other non-article items (e.g., conference proceedings) were removed. After these exclusions (*n* = 408), the final dataset consisted of 915 peer-reviewed journal articles, which formed the basis of the analysis (See Figure 1). 

1323 records; 189 non-eligible items (conference proceedings, book chapters, editorials, etc.) were excluded; and 219 duplicate entries were removed.

### 2.3. Data Analysis

Data analysis was conducted using the *bibliometrix 5.0* (Aria and Cuccurullo 2017) in *R* programming (version 4.4.3) a widely recognized tool for comprehensive bibliometric evaluation (Moral-Muñoz et al. 2020). The analyses encompassed multiple dimensions of scholarly activity, including the study (Elias 2019) productivity and impact metrics, collaboration networks, and conceptual/thematic structures. The results are reported in alignment with the three research questions (RQ1–RQ3), consistent with best-practice recommendations that emphasize transparent reporting of bibliometric procedures (Donthu et al. 2021).

- Performance Analysis

To assess research growth and productivity (RQ1), descriptive statistics and citation metrics (h-index, g-index, m-index) were applied (Ellegaard and Wallin 2015; Hirsch 2005; Egghe 2011). Annual publication output, author productivity, and journal/institutional contributions were mapped, while citation-based indicators were used to evaluate scholarly impact.

- Science Mapping

To examine the social and intellectual structure of the field (RQ2), co-authorship, institutional and country collaboration networks, and co-citation analyses were performed (Leydesdorff and Wagner 2008; Zupic and Čater 2015). These techniques identify collaboration patterns, geographic distribution of research partnerships, and shared theoretical foundations that underpin SEL scholarship.

- Thematic Analysis

To capture the conceptual structure and its evolution (RQ3), co-word based thematic mapping was conducted using Bibliometrix. Author keywords and Keywords Plus were combined as the units of analysis, with a minimum frequency threshold of five. Clusters were generated algorithmically using Callon’s centrality–density framework (Callon et al. 1991). This approach positions themes in a two-dimensional space where centrality measures the degree of a cluster’s interaction with other clusters (its importance in the overall network), and density measures the internal cohesion of the cluster (its development and internal consistency).

Based on these metrics, themes are classified into four categories:

- Motor themes (high centrality, high density): both conceptually central and well developed.
- Niche themes (low centrality, high density): internally strong but more peripheral.
- Emerging/declining themes (low centrality, low density): marginal, either nascent or waning.
- Basic themes (high centrality, low density): widely connected but less internally developed.

Importantly, classification is algorithmic rather than manual: terms such as gender may appear as motor themes if they are frequently used and strongly connected with other clusters, even if this may seem unexpected at first glance. This ensures transparency and reproducibility, as theme assignment reflects structural properties of the keyword co-occurrence network rather than subjective interpretation.

For thematic evolution, the corpus was divided into two periods (1983–2014 and 2015–2025). Evolution was mapped by tracing the continuity, transformation, or disappearance of clusters across periods, using Sankey-type visualizations to show how earlier clusters evolved (e.g., teacher into professional development), remained stable (e.g., early intervention), or declined (e.g., theory of mind).

## 3. Results

### 3.1. Performance Analysis of SEL Research in Primary Education Contexts

This section addresses RQ1 by presenting a bibliometric performance analysis that assesses the productivity and scholarly impact of authors, journals, institutions and countries using quantitative indicators of publication and citation metrics.

#### Citation and Publication Related Metrics

**Figure 2:**
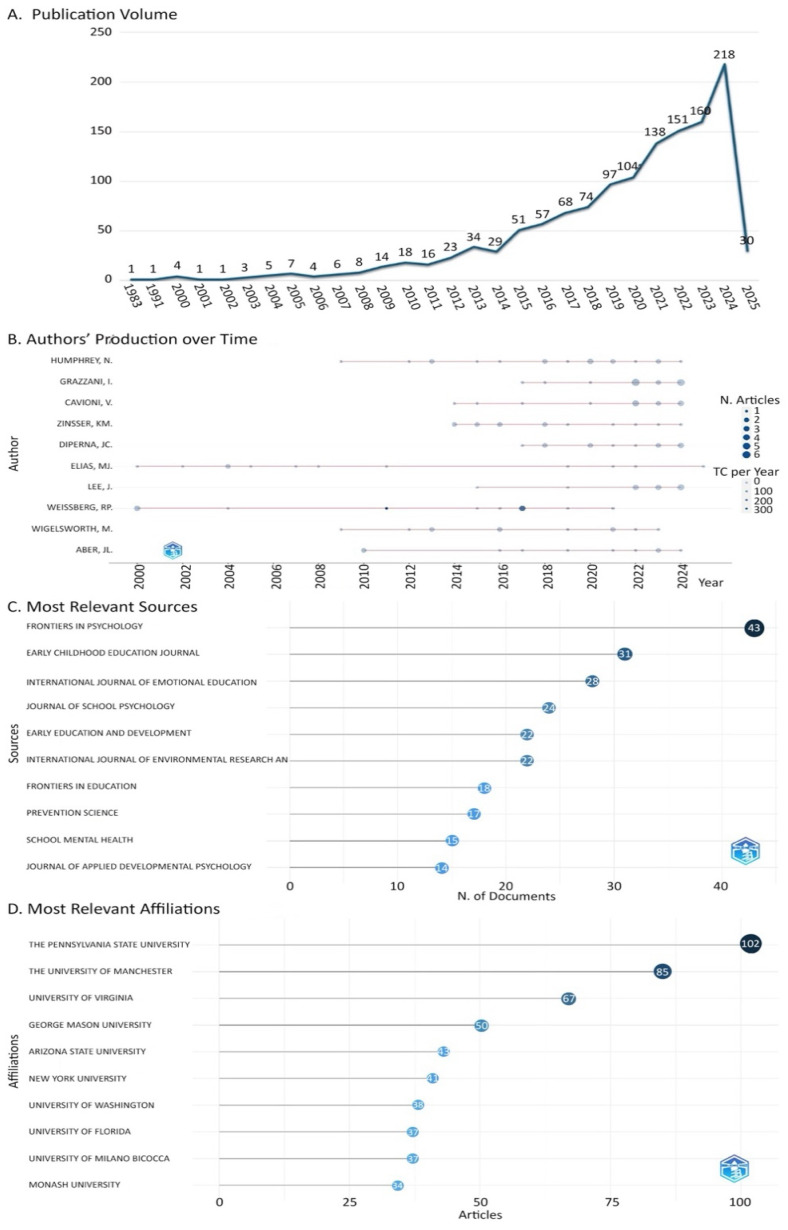
Overview of productivity and output in SEL research. (**A**) Annual publication trends from 1983 on. (**B**) Ten most prolific authors by total number of publications. (**C**) Ten leading journals ranked by publication count. (**D**) Top ten institutions contributing the highest number of publications.

The publication trajectory of SEL research shows a gradual increase beginning in 1983, with a marked surge after 2015. This growth intensified in the 2020s, reaching a peak of 218 articles in 2024, signaling heightened academic interest in the field (Figure 2A). As an indicator of research productivity, publication counts highlight leading contributors in the field. As shown in Figure 2B, Humphrey is the most prolific author in the field, with 18 publications, followed by Grazzani and Cavioni (16 each), and Zinsser (13). On the journal level, SEL studies have been disseminated through 582 different journals, reflecting a wide disciplinary interest. Figure 2C presents the ten most productive journals, led by Frontiers in Psychology (*n* = 43), followed by the Early Childhood Education Journal (*n* = 31) and the International Journal of Emotional Education (*n* = 28). Institutional output is equally notable. A total of 1398 institutions has contributed to SEL research within the primary education context. Figure 2D highlights the top ten institutions, where The Pennsylvania State University leads with 102 publications, followed by The University of Manchester (*n* = 85) and The University of Virginia (*n* = 67). Geographically, SEL research shows global engagement, spanning 73 countries. As summarized in Table 2, the United States emerges as the dominant contributor with 1419 articles, followed by Anglo-Saxon countries such as Australia (*n* = 152) and the United Kingdom (*n* = 144). Other key contributors include Canada (*n* = 96), Portugal (*n* = 91), China (*n* = 86), and Spain (*n* = 58), which represent a moderate level of production, while Finland (*n* = 35), Turkey (*n* = 31), the Netherlands (*n* = 28), and Greece (*n* = 27) appear with lower numbers of publications. These patterns confirm the central role of the U.S. but also show that SEL research is increasingly attracting contributions from a range of other countries.

According to Table 3, the citation metrics reveal distinct patterns of author impact and visibility. Weissberg, with 7659 citations, has significant scholarly recognition. However, his g-index of 12, and low m-index of 0.462, suggest limited annual citation growth, indicating fewer recent high-impact contributions. In contrast, Humphrey demonstrates a higher impact, with a g-index of 18 surpassing his h-index of 10, reflecting a few exceptionally well-cited publications that have garnered considerable academic attention. Grazzani, an emerging scholar who began publishing in 2017, shows a promising impact with an h-index (10), g-index (16), and m-index (1.111). This combination of a strong g-index and high m-index highlights her ability to produce influential work within a short time. On the other hand, Elias and Greenberg, despite their long careers, display relatively low productivity, with m-indices of 0.346 and 0.348, indicating slower citation growth over time.

According to the citation metrics in Table 4, distinct patterns of journal impact are evident. Early Education and Development, the International Journal of Emotional Education, and the Journal of School Psychology share the highest h-index values (13), reflecting their substantial and sustained contributions to the field. The Journal of School Psychology distinguishes itself further with the highest g-index (24), indicating a strong concentration of highly cited articles. While it has maintained consistent influence since its inception, with 493 citations from 16 articles, Early Education and Development shows a slightly higher g-index (21) and m-index (0.733), pointing to faster citation growth since 2010. In terms of the m-index, the International Journal of Environmental Research and Public Health leads with 1.286 and Frontiers in Psychology also demonstrates notable influence with a high m-index of 1, based on 43 publications since 2014.

As of February 2025, 1323 publications had been cited on SEL in primary schools. Leading this body of work, Durlak et al. (2011) remains the most influential, with 4783 global citations and 247 local citations, providing a foundational meta-analysis of the effectiveness of school-based SEL interventions. Taylor et al. (2017) follow, providing critical insights into the long-term impact of these programs, reflected in their 1218 global citations and 94 local citations. Mental health promotion is a recurring theme, with Weare and Nind (2011) receiving 599 global citations for their evidence-based evaluation of school-based mental health initiatives. In the area of applied interventions, Schonert-Reichl et al. (2015) stand out with 499 global citations, demonstrating the effectiveness of mindfulness-based approaches in improving children’s social-emotional and cognitive development. Furthermore, Domitrovich et al. (2017), with 446 global and 33 local citations, highlights the essential role of social-emotional competence in fostering resilience and reducing risk factors. Recent research includes Jagers et al. (2019), introducing transformative SEL for equity (327 citations), and Schonert-Reichl (2017), which focuses on teacher roles in SEL (312 citations). A comprehensive list of top-cited articles is provided in Table 5.

### 3.2. Science Mapping of SEL Literature in Primary Education Contexts

This section addresses RQ2 by using science mapping techniques to explore the social and intellectual structure of SEL literature in primary education.

#### 3.2.1. Collaboration Networks

**Figure 3:**
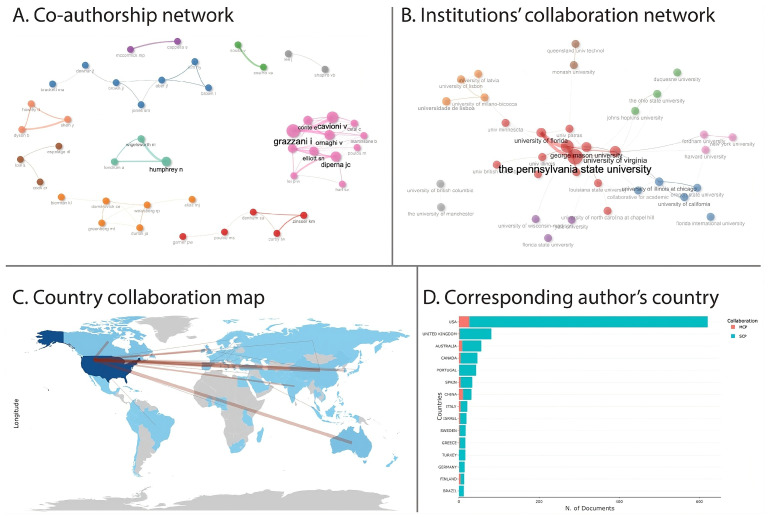
Collaboration networks. (**A**) Co-authorship network. (**B**) Collaboration network of institutions. (**C**) Collaboration of countries in publications. (**D**) Distribution of publications by corresponding author’s country. Note. SCP = Single-country publications, MCP = Multiple-country publications.

Figure 3A illustrates the co-authorship network of 45 authors engaged in SEL research, offering insights into the field’s social structure. The analysis reveals 10 distinct clusters, each representing varying levels of collaboration and thematic focus. Darker colors in the network indicate stronger co-authorship ties. Grazzani and Cavioni stand out as central figures within a densely connected cluster, marked by frequent collaboration. Humphrey also maintains a smaller but cohesive network, while close associations among Greenberg, Weissberg, and Domitrovich, as well as among Jones, Brown, Aber, and Kim, highlight enduring scholarly partnerships. In contrast, more isolated clusters, including those formed around Denham, Zinsser & Curby, Espelage & Cook, and Lee & Shapiro point to specialized or emerging contributions within the broader SEL research landscape.

Figure 3B illustrates the institutional collaboration network in SEL research, with 35 co-authorships across eight clusters. The red cluster dominates 13 inter-institutional links, where The Pennsylvania State University serves as a central hub, collaborating with 12 institutions and demonstrating its leadership in SEL research. Strong connections between The Pennsylvania State University, Arizona State University, the University of Florida and the University of Virginia further emphasize influential partnerships. In contrast, smaller clusters suggest independent or emerging research efforts. The University of Manchester and the University of British Columbia operate with limited co-authorship links, while Monash University and Queensland University of Technology reflect regional collaboration, indicating localized contributions to SEL research.

Figure 3C,D present the geographical distribution of corresponding authors and the collaborative landscape in SEL research, encompassing contributions from 73 countries. The USA leads the field with 621 articles and 26 multi-country publications (MCPs), forming extensive partnerships, particularly with China (10 collaborations) and Australia (7 collaborations). Other English-speaking countries, including the UK (81 articles), Australia (56), and Canada (46), also demonstrate strong engagement in MCPs. In Europe, Portugal (43) and Spain (33) emerge as prominent contributors, while China stands out in Asia with the highest MCP ratio (32.3%, 10 MCPs), reflecting its active global collaboration. Conversely, countries like Sweden (17), Greece (16), Turkey (16), Germany (14), and Brazil (12) primarily produce single-country publications (SCPs), indicating a more localized research focus and potential for future international engagement. The prevalence of SCPs across most countries suggests a national focus in SEL research. However, international collaboration remains significant, with 105 instances of cross-border partnerships identified. The USA leads in international ties with 72 collaborations across 31 countries, followed by strong bilateral connections between Italy (11 collaborations), Portugal (10 collaborations), and other European nations. While some regions, particularly in Africa and South America, exhibit fewer international links, this gap highlights opportunities to enhance collaborative networks and diversify SEL research contributions.

#### 3.2.2. Citation Network(s)

**Figure 4:**
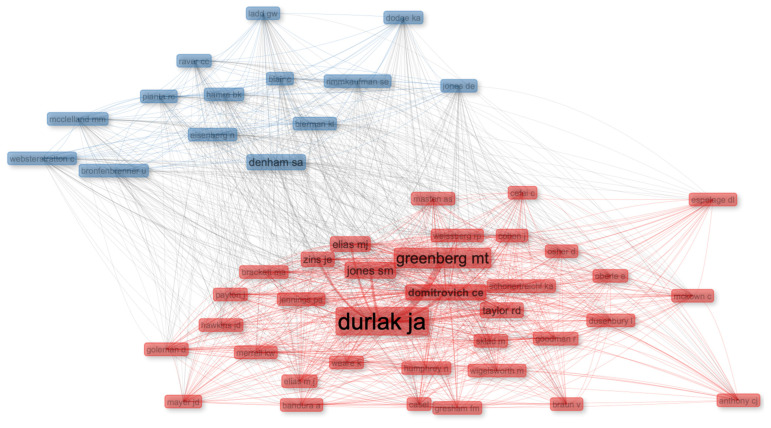
Co-citation network analysis.

The co-citation network analysis of 50 key sources further clarifies the intellectual architecture underpinning SEL research. As shown in Figure 4, the field is anchored by a dense core of foundational scholars—such as Durlak, Greenberg, Elias, Bandura, Goleman, Domitrovich, Jones, Jennings, Humphrey—whose work collectively shapes the theoretical and empirical basis of SEL. This tightly connected red cluster reflects consensus around key concepts like social-emotional competence, intervention effectiveness, and resilience-building within educational contexts. In contrast, the more dispersed blue cluster without a central figure, featuring scholars like Denham, Dodge, Hamre, Rimm-Kauffman, and Pianta, signals the diversification of SEL scholarship into emerging and specialized domains, including early childhood emotional development, teacher-student dynamics, and context-driven program adaptations.

### 3.3. Research Trends, Major Themes, and Future Directions

This section addresses RQ3 by examining the development of SEL research themes in primary education. Using keyword frequency and trend analyses alongside thematic clustering techniques, it highlights how the field’s thematic focus and conceptual landscape have shifted across different publication periods.

#### 3.3.1. Keyword and Trends Analysis

**Figure 5:**
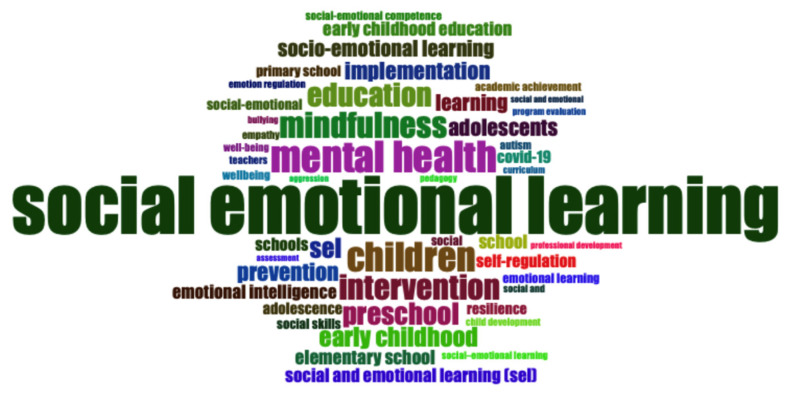
Top 50 most frequently used author keywords.

Keyword and trend analyses highlight how SEL research is conceptually structured and thematically diverse. Across the dataset, 2831 distinct keywords were identified, underscoring the field’s breadth. As shown in Figure 5, the dominant cluster is social and emotional learning, unifying multiple variants and appearing 744 times, which confirms its centrality as both a construct and research label. Other prominent clusters include education and schooling (*n* = 207), mental health and well-being (*n* = 167), and intervention/implementation (*n* = 145), reflecting strong emphases on educational contexts, psychological health, and program delivery. Developmental lenses also stand out, particularly early childhood/preschool (*n* = 108) and adolescence/adolescents (*n* = 56), alongside clusters related to children (*n* = 72) and social skills (*n* = 72). In addition, themes such as emotional intelligence (*n* = 67), teachers and professional development (*n* = 29), academic achievement (*n* = 17), autism (*n* = 17), bullying (*n* = 14), and aggression (*n* = 13) capture the field’s concern with cognitive, behavioral, and contextual dimensions. To improve clarity, Supplementary Table S1 presents the unified keyword categories and their aggregated frequencies, complementing Figure 5 and making the organization of terms transparent.

**Figure 6:**
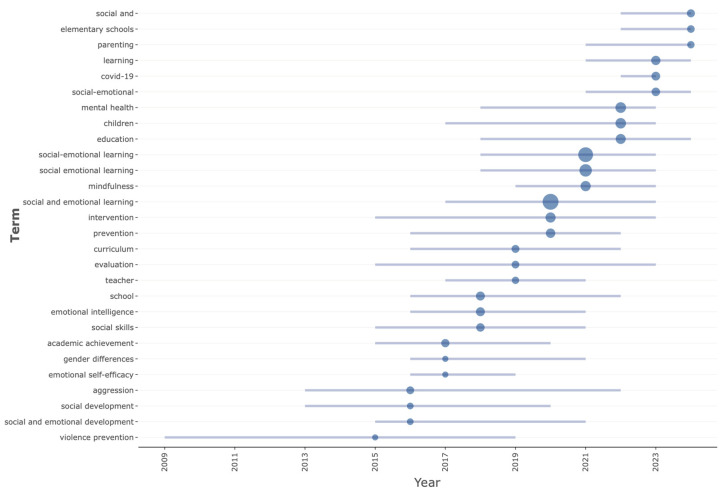
Temporal distribution of emerging trend topics across publications.

To better understand the development of the thematic structure, time frame was divided into three distinct periods: 2009–2014, 2015–2019, and 2020–2024. During the first period, key terms such as violence prevention, aggression, social development, emotional self-efficacy, and gender differences were most prominent, reflecting an early focus on behavioral and emotional development within SEL research. In the second period, terms like social and emotional learning (including its variants), mindfulness, social skills, emotional intelligence, intervention, curriculum, teacher, and school gained prominence, indicating a shift toward conceptual expansion and applied practices. The most recent period shows the emergence and increasing frequency of terms such as COVID-19, parenting, elementary schools, learning, education, mental health, and children, highlighting the growing emphasis on systemic, school-wide approaches and the impact of global contexts (see Figure 6).

#### 3.3.2. Thematic Clustering and Evolution of Research Topics

**Figure 7:**
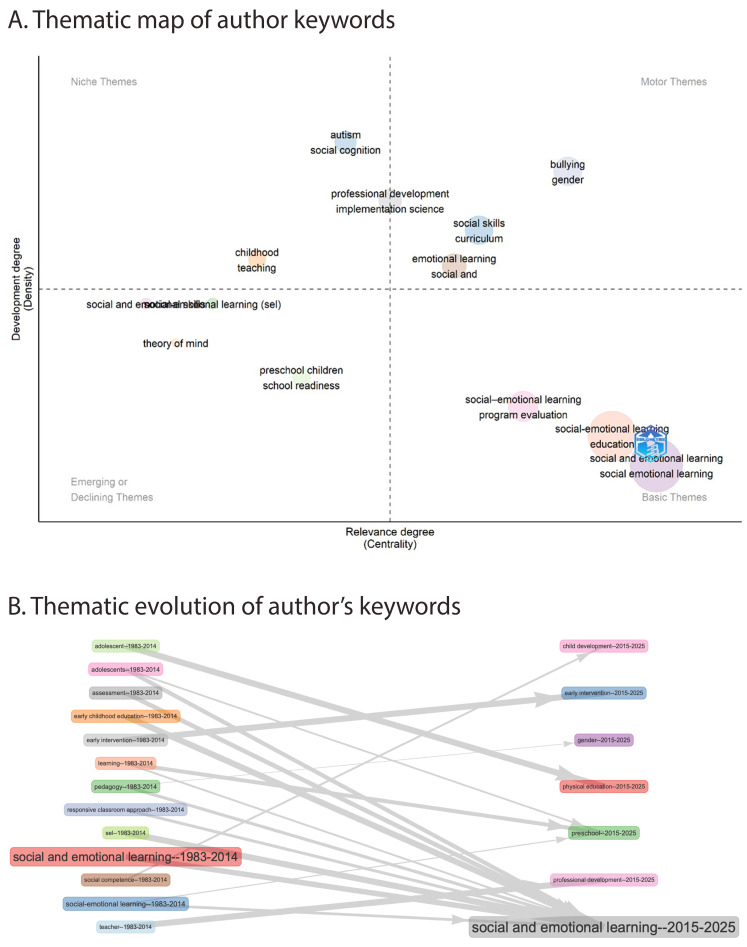
Thematic mapping. (**A**) Thematic map illustrating clusters of author keywords. (**B**) Thematic evolution of author keywords across two time periods.

This analysis focuses on the conceptual organization and structural development of SEL research over time. A categorical thematic mapping approach was applied to identify central, peripheral, and evolving themes and their interrelationships. As shown in Figure 7A, a total of 13 thematic clusters were identified and categorized into motor themes, niche themes, emerging or declining themes, and basic themes. The densest clusters and their high-frequency keywords are presented in Supplementary Table S2. As these clusters were algorithmically derived, categories were not created manually but reflect the centrality–density structure of the keyword network.

Motor themes, which are both highly central and internally coherent, include “social and emotional learning”, “education”, “school skills”, “bullying”, and “gender”. Among these, the densest cluster is “social skills”, comprising keywords such as “curriculum”, “life skills”, and related terms. Niche themes are defined as specialized and focused but less connected within the broader thematic structure. In this study, niche themes include “autism”, “social cognition”, “professional development”, “implementation science”, “childhood”, and “teaching”. The densest cluster is “professional development”, with keywords like “implementation science” and “early childhood education and care”. Emerging or declining themes are characterized by low centrality and density, indicating areas either gaining visibility or decreasing in relevance. Identified themes in this category include “theory of mind”, “preschool children”, and “school readiness”, with the densest cluster being “preschool children”. Basic themes are defined by high centrality and low density, indicating that they are closely connected to other themes but remain less developed or unified internally. In this study, “program evaluation” and “social-emotional learning” are categorized as basic. The densest cluster there is “social-emotional learning”, represented by terms such as “mental health”, “children”, “intervention”, etc.

The thematic evolution structure is presented in Figure 7B and summarized in Supplementary Table S3, illustrating shifts in research focus between the periods 1983–2014 and 2015–2025. The findings indicate that “social and emotional learning” remained a central and overarching theme across both periods. While “early intervention” also persisted as a stable theme, other terms shifted over time: “teacher” evolved into “professional development”, “social competence” broadened into “child development”, and “adolescents” became linked with “physical education”. Additionally, “gender” emerged as a distinct new theme in the later period, reflecting the growing diversification of SEL-related research foci.

## 4. Discussion

Mapping the development of SEL research in primary education provides critical insight into the growth, focus and emerging priorities of the field. This study offers a broader and more nuanced understanding than previous bibliometric analyses, drawing on 915 peer-reviewed documents-significantly more than included in previous reviews (e.g., Ağırkan and Ergene 2021: *n* = 321; Haruna and Isa 2024: *n* = 64; Manjarres et al. 2023: *n* = 340). In addition to covering a longer publication window (1983–2025), this study enhances representativeness by integrating records from both Web of Science and Scopus and employing a more comprehensive keyword strategy. Beyond descriptive metrics, the study contributes a layered analysis by incorporating trend topic modeling and thematic evolution mapping, providing deeper insights into conceptual development and structural shifts in SEL scholarship over time. The following discussion revisits each research question, focusing on what the data reveal about the field’s trajectory, persistent gaps, and the contextual dynamics that influence global knowledge production in SEL.

### 4.1. The Performance Analysis

Performance analysis provides a descriptive overview of the scientific productivity and scholarly impact of contributors to a research field, offering insights into who produces knowledge, where it is published, and how that knowledge circulates. In the case of SEL research, this analysis reveals a pronounced and accelerating trajectory, particularly from the mid-2010s onward. While early conversations about children’s social development in schools date back to the 1980s, the concept of SEL was formally introduced in 1994, when a group of educators, researchers, and advocates convened under the Fetzer Institute to address the “missing piece” in education (Elias et al. 1997). This collaboration led to the founding of the CASEL and the coining of the term “social and emotional learning” (Elbertson et al. 2009). Subsequent milestones, including the first formal guidance on school-based SEL programs in 2003 and a 2004 synthesis linking SEL to academic success, laid the foundation for a robust empirical field (CASEL 2025a). A pivotal shift occurred with Durlak et al.’s (2011) landmark meta-analysis, which demonstrated an 11 percentile-point improvement in academic performance among students exposed to SEL interventions. Since then, the field has experienced rapid growth, reaching a record 218 publications in 2024. This expansion reflects mounting international recognition of SEL’s relevance not only for fostering academic success but also for enhancing broader developmental outcomes. Empirical findings across diverse contexts increasingly support that students participating in SEL interventions exhibit improved social-emotional competencies, more positive attitudes, enhanced behavior, better peer relationships, and improved perceptions of school climate and safety relative to their peers in control conditions (Cipriano et al. 2023; Aygün and Taşkın 2022; Tubbs Dolan et al. 2022; Marsay 2022). Collectively, these results—documented in both high-income and low- and middle-income countries—have elevated SEL from an innovative educational supplement to a developmental and pedagogical necessity.

This upward trend in publication output is mirrored by the scholarly productivity of both foundational and emerging figures in SEL research. Early pioneers such as Weissberg, Elias, and Greenberg not only laid the conceptual groundwork for the field but also institutionalized SEL through the founding of CASEL and early school-based initiatives (CASEL 2025b). Their enduring influence is evident in continued high citation rates and their formative role in shaping SEL frameworks. However, the current research landscape reflects a generational and geographic broadening. Newer scholars such as Humphrey, Grazzani and Cavioni are among the most prolific, indicating a turn towards more contextual enquiry, asking not only what SEL is, but how to implement it effectively in different educational contexts and for whom. These scholars represent not just individual productivity, but also the institutional ecosystems that support SEL research. While U.S.-based institutions dominate in volume, and The Pennsylvania State University (Weissberg, Bierman) and the University of Virginia appears among the top producers, European hubs such as the University of Manchester (Humphrey), and the University of Milano-Bicocca (Grazzani, Cavioni) are particularly influential in shaping conceptual and implementation debates within the field. This institutional concentration maps onto broader national patterns. The United States remains the dominant contributor to SEL literature, producing the highest volume of research and serving as a historical and conceptual origin point for the field. U.S. leadership has been supported by robust philanthropic foundations (e.g., the Fetzer Institute) and national funding structures that prioritize SEL as an educational and public health concern (Yoder and Dang 2023). Similarly, in the United Kingdom, Australia, and Canada, the integration of SEL into national education strategies has facilitated government-sponsored program evaluations (e.g., Promoting and Supporting Mental Health and Wellbeing in Schools and Colleges) and cross-sector collaborations (e.g., Maloney et al. 2024). In the European context, SEL has gained policy-level traction through targeted funding by the European Commission. Initiatives co-funded by Erasmus+ Programmes exemplify this commitment by embedding mental health promotion and SEL into school systems across multiple countries (e.g., PROMEHS scientific publications and presentations). This regional investment underscores a growing recognition of SEL not merely as a pedagogical innovation but as a public health and equity strategy aligned with broader EU education goals (Cefai et al. 2018). China and Israel also appear among the top ten contributing countries, indicating their emerging contributions to SEL research, though on a more modest scale compared to the U.S. and European hubs.

The diversification of SEL research is further underscored by the breadth of its publication landscape. With contributions from 582 journals, SEL scholarship spans developmental psychology, early childhood education, public health, and educational policy, reflecting its wide interdisciplinary appeal. While Frontiers in Psychology, Early Childhood Education Journal, and International Journal of Emotional Education lead in terms of publication volume; Early Education and Development, the International Journal of Emotional Education, and the Journal of School Psychology emerge as the most impactful outlets based on citation metrics. This pattern reflects the field’s emphasis on evidence-based, intervention-focused research that bridges psychological theory with practical applications in educational settings. Such scholarship reinforces the strategic role of schools as delivery systems for SEL, offering consistent, inclusive, and developmentally aligned environments for large-scale implementation (O’Brien et al. 2023; Greenberg and Abenavoli 2017).

This scholarly trajectory is anchored by a small set of highly cited publications that have shaped the evidence base of the field. Durlak et al.’s (2011) meta-analysis remains the most influential work to date, demonstrating the robust academic and developmental benefits of SEL interventions. Building on this foundation, Taylor et al. (2017) extended the analysis to long-term outcomes, while subsequent studies by Weare and Nind (2011), Schonert-Reichl et al. (2015), and Domitrovich et al. (2017) diversified the field’s scope to include mental health promotion, mindfulness, and resilience. These contributions collectively define the empirical core of SEL and continue to guide both implementation and policy.

### 4.2. The Science Mapping

The collaboration network analysis highlights both the strengths and limitations of social structures within SEL research. At the author level, ten distinct clusters reveal a pattern of concentrated collaboration around key figures such as Grazzani and Cavioni in Europe, and Humphrey in the UK, alongside enduring partnerships among foundational scholars like Greenberg, Weissberg, and Domitrovich linked to CASEL initiatives. These dense networks suggest that while SEL research benefits from established scholarly communities, more specialized or emerging areas, such as teacher beliefs (Zinsser, Curby) and bullying prevention (Espelage, Cook), tend to operate within smaller, less connected groups. This pattern extends to institutional collaboration, where The Pennsylvania State University anchors a dominant network, reflecting its leadership in SEL scholarship. However, universities such as Manchester and British Columbia exhibit more localized collaboration, indicating that institutional partnerships remain unevenly distributed across regions. Geographically, SEL research is predominantly driven by English-speaking countries, with the USA leading both in research output and international partnerships—particularly with China and Australia. China’s high multi-country publication ratio underscores its growing role in global academic networks, while European countries like Portugal and Italy show promising collaboration patterns within the region. The prevalence of single-country publications across much of Europe, Asia, and South America points to a persistent national focus in SEL research efforts.

While promising, this global expansion also accentuates deep-seated inequities in collaborative participation. The dominance of English-speaking countries and select European hubs is in stark contrast to the limited involvement of African and Latin American countries, as well as other low- and middle-income countries (LMICs). This imbalance reflects not only academic preference, but also structural disparities in research capacity, infrastructure, and equitable access to funding opportunities. This asymmetry is an example of the ongoing North–South divide in global knowledge production (Tikly 2011; Unterhalter 2012), in which scholarly influence and agenda-setting are concentrated in well-resourced regions. This entrenched divide poses significant challenges for the future of SEL scholarship (Coetzee and Loades 2025). Without intentional efforts to decentralize research leadership and foster inclusive, transnational collaboration, SEL frameworks risk perpetuating Western-centric models that fail to address the socio-cultural realities of underrepresented regions. Recent evidence illustrates both the scarcity and the promise of SEL in LMICs: in Turkey, school-based programs have shown positive socio-emotional outcomes (Ağırkan and Ergene 2021; Aygün and Taşkın 2022); in Latin America, SEL initiatives have demonstrated adaptability and impact in low-resource environments (Tubbs Dolan et al. 2022); and in South Africa, scholars have stressed the urgent need for SEL skills interventions, particularly since COVID-19 (Marsay 2020a, 2020b; Marsay et al. 2021). LMICs are home to the majority of the world’s children, many of whom face conflict, adversity and heightened vulnerability in socio-emotional development (Kamøy et al. 2021; Khan et al. 2023; Şahin et al. 2021; Yang et al. 2019). Therefore, this gap is not just academic, but also a pressing issue of global educational equity. Beyond this, the gap is also structural, as major bibliometric databases such as WoS and Scopus disproportionately index English-language and high-income country journals (Archambault et al. 2006; Mongeon and Paul-Hus 2016). Consequently, contributions from LMICs or non-English outlets may be underrepresented in bibliometric datasets, even if SEL initiatives are active locally. Recognizing these limitations reinforces the urgency of fostering more geographically inclusive and culturally responsive SEL scholarship. Expanding collaborative networks and rethinking funding priorities are essential steps not only to diversify academic discourse, but also to ensure that SEL fulfills its potential as a globally relevant, culturally responsive tool for fostering resilience, well-being and educational opportunity.

The co-citation network analysis reveals the cohesive yet evolving intellectual architecture of SEL research, where foundational theories, empirical evidence, and practical applications converge. At the core lies a dense cluster of influential scholars—Durlak, Weissberg, Taylor, Greenberg, Domitrovich, Jones, and Jennings—whose frequent collaborations have shaped the foundation of evidence-based interventions, systemic implementation strategies, and teacher-centered SEL approaches. Through large-scale meta-analyses, Durlak, Weissberg, and Taylor have demonstrated the long-term academic, social, and emotional benefits of universal, school-based SEL programs (Durlak et al. 2011; Taylor et al. 2017). Greenberg has extended this perspective by positioning SEL as a public health strategy, emphasizing resilience and mental health promotion through school-wide frameworks (Greenberg et al. 2017). Domitrovich’s contributions further highlight the critical role of implementation fidelity and scalability in ensuring program effectiveness, exemplified by her development of the Preschool PATHS^®^ curriculum, which provides a structured, evidence-based approach to fostering social-emotional skills in early childhood education (Domitrovich et al. 2007). Simultaneously, Jones has advanced SEL through the creation of structured frameworks and equity-focused, scalable interventions—such as SEL Kernels—ensuring that implementation strategies remain both practical and adaptable across varied educational settings (Jones et al. 2017). Jennings enriches this core by emphasizing teacher social-emotional competence and mindfulness-based interventions, demonstrating how educator well-being shapes classroom climates and student outcomes (Jennings and Greenberg 2009). The theoretical backbone of this cluster is reinforced by Bandura’s social cognitive theory (Bandura 1977) and Goleman’s emotional intelligence framework (Goleman 1995), which continue to inform how social-emotional competencies are conceptualized and applied. Humphrey has also contributed significantly to large-scale evaluations, implementation variability, and policy-driven SEL research, particularly within the UK, reflecting a shift towards scalable, context-sensitive solutions (Humphrey 2023).

In contrast, the more dispersed blue cluster reflects the diversification of SEL research into specialized domains. Denham’s pioneering work has shaped the understanding of emotional development in early childhood, highlighting the critical role of emotional competence, socialization, and parental influences in fostering social adaptation and school readiness (Denham 1998; Denham and Brown 2010). Dodge’s seminal work on social information processing and aggression has been pivotal in linking SEL frameworks to behavioral regulation, conflict resolution, and the understanding of proactive and reactive aggression in children’s social adjustment (Crick and Dodge 1994; Dodge and Coie 1987). Meanwhile, Rimm-Kaufman’s research emphasizes how classroom ecology, teacher-child interactions, and self-regulation contribute to adaptive behaviors and smooth transitions in early education (Rimm-Kaufman and Pianta 2000; Rimm-Kaufman et al. 2014). Hamre and Pianta, on the other hand, have focused on systematically defining and measuring classroom quality, demonstrating that strong teacher-child relationships and balanced instructional-emotional support are critical predictors of students’ long-term academic and social-emotional success. Their contributions, including the widely adopted CLASS framework, have been instrumental in operationalizing effective socio-emotional learning environments (Hamre and Pianta 2001; Pianta et al. 2008).

### 4.3. Research Trends, Major Themes, and Future Directions

Keyword analysis reveals what topics dominate SEL discourse and when they emerge, while thematic clustering uncovers how these topics interconnect conceptually and evolve over time within the field. The results from my dataset of 2831 unique keywords show a clear temporal shift in the focus of SEL research over the last 15 years.

To capture the evolving thematic structure of SEL research, the data were analyzed across three distinct time periods with keyword analysis: 2009–2014, 2015–2019, and 2020–2024. In its early phase (2009–2014), SEL research predominantly addressed behavioral challenges and emotional development, with frequent keywords such as violence prevention, aggression, emotional self-efficacy, and social development. These patterns reflect a foundational interest in mitigating problematic behaviors and fostering intrapersonal regulation, goals rooted in the field’s origins in psychology and youth risk prevention. In the second period (2015–2019), the field experienced a notable conceptual expansion. Keywords such as schools, teacher, curriculum, intervention, and evaluation became more prominent, signaling a shift toward systemic implementation within formal education settings. This evolution reflects a growing recognition of schools not only as instructional spaces but also as strategic platforms for both delivering SEL and monitoring student well-being (Oberle and Schonert-Reichl 2017). Importantly, the increasing emphasis on teachers as implementers and co-regulators of SEL underscores the field’s movement beyond isolated interventions toward integrated, curriculum-embedded approaches that are sustained through everyday classroom interactions. Between 2020 and 2024, the data reveal a further evolution in thematic focus. Prominent keywords such as mental health, parenting, elementary schools, and COVID-19 reflect SEL’s expanding alignment with broader efforts to foster resilience in the face of global crises. The COVID-19 pandemic, in particular, heightened awareness of the psychological impacts of prolonged isolation and school closures on children and adolescents (Wang et al. 2020), reinforcing SEL’s foundational premise: that all children inevitably encounter adversity and thus require core competencies to navigate stress, adapt, and flourish (Lopes and Salovey 2004). The increased salience of elementary schools as a keyword further validates this study’s emphasis on primary education, indicating a shift toward the systemic integration of SEL in primary school contexts. Simultaneously, family engagement has emerged as a critical reinforcing domain. Research underscores that when schools and families operate in partnership—aligned through shared goals and mutual responsibility—SEL initiatives become more coherent and sustainable (Albright and Weissberg 2010; Garbacz et al. 2015). These findings collectively demonstrate that SEL research has moved from individual-level constructs to broader, school-wide, and context-sensitive perspectives.

The thematic clustering and evolution analysis provides a deeper understanding of how SEL research has become increasingly structured and differentiated over time. By categorizing themes into motor, niche, emerging/declining, and basic types, the field’s conceptual architecture becomes more visible. The identification of motor themes such as social and emotional learning, education, school skills, bullying, and gender underscores their conceptual centrality and enduring practical significance in SEL research. Among them, social skills emerged as the densest cluster, reflecting their dual role as foundational constructs and actionable targets in school-based interventions. Rooted in theoretical traditions from notion of social intelligence to the model of emotional intelligence, these skills embody the intertwined nature of social and emotional development. Core competencies such as social awareness and relationship skills—empathy, cooperation, and conflict resolution—are not only observable and teachable, but also directly linked to academic engagement and broader life outcomes (Greenberg et al. 2017). Their thematic prominence affirms SEL’s emphasis on fostering adaptive, transferable behaviors that support both personal growth and social cohesion.

Among the niche themes identified in this study, professional development emerged as the most prominent, frequently appearing alongside implementation science, literacy, and early childhood education and care. While these themes are less central to the overall field structure, their prominence signals an increasing scholarly focus on the practical conditions that shape SEL effectiveness. The consistent association of professional development with implementation science reflects the field’s gradual shift toward recognizing that program success depends heavily on teachers’ preparedness, confidence, and capacity to adapt SEL approaches to diverse contexts (Alemdar and Anılan 2020; Adi et al. 2007). This is consistent with previous findings that highlight the decisive role of teacher engagement, knowledge, and delivery style in shaping implementation quality and, ultimately, student outcomes (Humphrey et al. 2018; Macklem 2020). This growing emphasis on the “how” of SEL—not just the “what”—marks a maturation of the field toward implementation-sensitive and practice-informed inquiry.

Emerging or declining themes—such as theory of mind, preschool children, and school readiness—were characterized by low density and centrality, marking them as either nascent or waning areas of inquiry within the broader SEL landscape (Kaiser and Kuckertz 2023). Notably, preschool children emerged as the densest cluster within this category and was also identified in both the keyword frequency and thematic evolution analyses. This dual presence suggests that, despite its current peripheral position on the thematic map, preschool children is more likely an emerging rather than declining focus within SEL research. This aligns with a growing body of literature emphasizing early childhood as a critical period for social-emotional development, with interventions during this stage demonstrating significant benefits for behavioral, academic, and neurobiological outcomes (McClelland et al. 2017; Schmitt et al. 2015). Moreover, longitudinal research confirms that early SEL competencies strongly predict later educational attainment, well-being, and public health outcomes (McClelland et al. 2013; Jones et al. 2015). Accordingly, while preschool children currently occupy a peripheral position, its identification as a dense emerging theme suggests it represents a promising research frontier that merits deeper investigation and sustained investment.

In my analysis, the basic themes quadrant, characterized by high centrality but low density, positions “social-emotional learning” (SEL) as the densest and most interconnected cluster. This pattern affirms SEL’s foundational role across the field, encompassing an expansive constellation of related keywords that can be meaningfully organized into four interlinked clusters: (1) core SEL constructs (e.g., emotional intelligence, resilience, mindfulness, emotion regulation); (2) target populations and educational contexts (e.g., children, adolescents, teachers, school settings); (3) intervention strategies and delivery models (e.g., universal prevention, school-based interventions, pedagogy, assessment, implementation); and (4) systemic and contextual challenges (e.g., mental health, trauma, COVID-19, school climate). This thematic breadth reflects SEL’s extensive influence across educational, health, and policy domains (Jones and Doolittle 2017), yet its classification as a basic theme highlights a persistent lack of internal cohesion among these clusters. While SEL frameworks proliferate globally—over 136 identified (Berg et al. 2017)—they remain shaped by diverse disciplinary logics, leading to conceptual ambiguity, inconsistent program models, and challenges in cross-contextual adaptation (Cipriano et al. 2023). The findings echo these by showing that although SEL dominates the field’s structural map, the connections between its core competencies, delivery processes, target populations, and systemic challenges often remain fragmented. Key subdomains such as implementation, assessment, and pedagogy emerge as prominent yet still loosely connected elements, underscoring the need for more integrated approaches that explicitly link “what” SEL teaches, “how” it is delivered, and “for whom” it is designed (Cipriano et al. 2023). Moreover, the clustering of keywords related to systemic challenges, including mental health, trauma, and COVID-19, reinforces SEL’s positioning as a lever for resilience-building and educational equity, particularly in the wake of global crises (Barker and Franklin 2017). However, without stronger alignment between constructs, delivery models, and systemic contexts, SEL risks remaining conceptually diffused and operationally fragmented. Addressing these gaps will require intensified efforts to consolidate SEL taxonomies, advance implementation science, and foster interdisciplinary dialog that bridges theory, practice, and policy, especially as SEL becomes embedded within complex, whole-school and universal interventions.

## 5. Limitations

This study has several limitations. First, as with any bibliometric analysis, the scope of the findings is defined by the data sources used. Relying on WoS and Scopus ensured standardized metadata and reproducibility, but it also meant that some relevant SEL studies—particularly those indexed in other databases (e.g., ERIC, PsycINFO) or in gray literature (e.g., book chapters, reports, dissertations)—were not captured. This constraint is common in bibliometric research (Donthu et al. 2021; Moral-Muñoz et al. 2020) and should be considered when interpreting the results. Importantly, both WoS and Scopus disproportionately cover English-language and high-income country journals, while contributions from LMICs or non-English outlets may be underrepresented (Archambault et al. 2006; Mongeon and Paul-Hus 2016). As a result, SEL initiatives documented in local or non-indexed sources may not be fully visible in the bibliometric maps, and findings about global asymmetries must be interpreted in light of these database-related biases.

Second, the over-representation of U.S.-based publications (≈70% of the corpus) may have inflated the visibility of American institutions and networks, potentially obscuring contributions from other regions. To temper this effect, country-level outputs were reported using fractional counting and Table 1 was expanded to include the top 15 countries. Nonetheless, this asymmetry remains a structural feature of the field and is explicitly acknowledged in both the Discussion and Implications sections.

Finally, thematic mapping is sensitive to keyword selection and clustering algorithms. Although robustness checks were performed using bibliometrix defaults and widely adopted Callon centrality–density metrics, thematic boundaries may vary slightly with different parameter choices. Therefore, the thematic maps should be read as indicative of broad conceptual structures rather than as fixed or exhaustive taxonomies.

## 6. Implications for Research, Practice, and Policy

This bibliometric analysis revealed a U.S.-led core and regional clustering in Europe, as well as minimal contributions from LMICs. This highlights the ongoing asymmetries in collaboration. In terms of research, this suggests the need for equitable partnerships with LMICs and regional consortia to reduce single-country silos. Journals and funders can support transparency by providing simple disclosure statements on authorship geography and budget allocation. Future studies can also adopt interdisciplinary methodologies, combining insights from education, psychology and implementation science, and incorporate hybrid effectiveness–implementation designs to examine outcomes and context-specific adaptations in under-represented regions.

In practice, scaling up SEL requires culturally responsive and context-sensitive approaches, including bilingual protocols, clear reporting of local adaptations and implementation costs, and—where feasible—harmonization of outcome measures to contribute to the consolidation of SEL taxonomies. This would enhance comparability without losing local relevance.

At the policy level, it is essential to diversify funding priorities to include capacity building in LMICs. Journals can promote equity by adopting editorial policies such as open materials and code, and transparent reporting of collaboration structures. Ministries can also incentivize cross-country collaboration to ensure that SEL frameworks reflect diverse sociocultural realities. Diversifying funding priorities and improving indexing practices could also mitigate the over-representation of High income countries/English sources in global bibliometric datasets, thereby ensuring that SEL research from underrepresented regions gains greater visibility in the international evidence base.

## Figures and Tables

**Table 1:** Bibliometric analysis procedures across research questions (RQ1–RQ3).

The Analysis and Goal	Technique(s)	Purpose of Usage	Unit of Analysis	Output of Analysis
RQ1 Performance Analysis Mapping productivity and impact	Descriptive statistics; Citation metrics (h-index, g-index, m-index); Productivity counts	To assess research growth, productivity, and impact of authors, journals, institutions, and countries	Articles, Authors, Journals, Institutions, Countries	Annual publication output; cumulative growth; citation impact indicators; identification of key contributors
RQ2 Science Mapping Revealing social and intellectual structure	Co-authorship, Institutional and Country collaboration analysis; Co-citation analysis	To uncover social interactions, collaboration patterns, and intellectual foundations in the field	Authors, Institutions, Countries, Journals, References	Collaboration networks; identification of implicit research communities and intellectual schools of thought
RQ3 Thematic Analysis Revealing conceptual structure and evolution	Thematic clustering; Thematic evolution analysis	To detect dominant clusters, conceptual architecture, and emerging/declining themes over time	Author Keywords, Keywords Plus	Thematic map; thematic evolution diagrams; identification of conceptual structure and trend dynamics

*Source:* Adapted from Zupic and Čater (2015), Aria and Cuccurullo (2017), and Donthu et al. (2021).

**Table 2:** Countries’ scientific production.

	Country	Publications
1	USA	1419
2	Australia	152
3	UK	144
4	Canada	96
5	Portugal	91
6	China	86
7	Spain	58
8	Italy	46
9	Sweden	43
10	Israel	42
11	Germany	40
12	Finland	35
13	Turkey	31
14	Netherlands	28
15	Greece	27

**Table 3:** Author productivity in SEL research within primary education contexts.

	Author	h-Index	g-Index	m-Index	Total Citation	Total Article	Career Starting
1	Weissberg, R. P.	12	12	0.462	7659	12	2000
2	Denham, S. A.	10	10	0.625	932	10	2010
3	Grazzani, I.	10	16	1.111	284	16	2017
4	Humphrey, N.	10	18	0.588	360	18	2009
5	Cavioni, V.	9	13	0.75	221	13	2014
6	Elias, M. J.	9	12	0.346	270	12	2000
7	Zinsser, K. M.	9	13	0.75	389	13	2014
8	Coelho, V. A.	8	10	0.727	212	10	2015
9	Domitrovich, C. E.	8	10	0.8	1100	10	2016
10	Greenberg, M. T.	8	9	0.348	1107	9	2003

**Table 4:** Journal effectiveness in SEL research within primary education contexts.

	Journal	h-Index	g-Index	m-Index	Total Citation	Total Articles	Publication Starting
1	Early Education and Development	13	22	0.813	949	22	2010
2	International Journal of Emotional Education	13	20	0.813	406	28	2010
3	Journal of School Psychology	13	24	0.542	974	24	2002
4	Frontiers in Psychology	12	19	1	416	43	2014
5	Prevention Science	11	17	0.478	894	17	2003
6	Early Childhood Education Journal	10	21	0.526	447	31	2007
7	Psychology in the Schools	10	14	0.5	310	14	2006
8	International Journal of Environmental Research and Public Health	9	15	1.286	248	22	2019
9	School Psychology Quarterly	9	11	0.5	564	11	2008
10	Journal of Applied Developmental Psychology	8	14	0.381	416	14	2005

**Table 5:** Most cited articles in the reviewed publications.

	Articles		Global Citations	Local Citations
1	Durlak, J. A., Weissberg, R. P., Dymnicki, A. B., Taylor, R. D., & Schellinger, K. B. (2011). The impact of enhancing students” social and emotional learning: A meta-analysis of school-based universal interventions. *Child Development*, *82*(1), 405–432. https://doi.org/10.1111/j.1467-8624.2010.01564.x	(Durlak et al. 2011)	4783	247
2	Taylor, R. D., Oberle, E., Durlak, J. A., & Weissberg, R. P. (2017). Promoting positive youth development through school-based social and emotional learning interventions: A meta-analysis of follow-up effects. *Child Development*, *88*(4), 1156–1171. https://doi.org/10.1111/cdev.12864	(Taylor et al. 2017)	1218	94
3	Weare, K., & Nind, M. (2011). Mental health promotion and problem prevention in schools: What does the evidence say? *Health Promotion International*, *26*(suppl_1), i29–i69. https://doi.org/10.1093/heapro/dar075	(Weare and Nind 2011)	599	16
4	Schonert-Reichl, K.A., Oberle, E., Lawlor, M.S. et al. (2015). Enhancing cognitive and social–emotional development through a simple-to-administer mindfulness-based school program for primary school children: A randomized controlled trial. *Developmental Psychology*, *51*(1), 52–66. https://doi.org/10.1037/a0038454	(Schonert-Reichl et al. 2015)	499	24
5	Domitrovich, C.E., Durlak, J.A., Staley, K.C., & Weissberg, R. P. (2017). Social-emotional competence: An essential factor for promoting positive adjustment and reducing risk in school children. *Child Development*, *88*(2), 408–416. https://doi.org/10.1111/cdev.12739	(Domitrovich et al. 2017)	446	33
6	Kam, C.M., Greenberg, M.T. & Walls, C.T. (2003). Examining the role of implementation quality in school-based prevention using the PATHS curriculum. *Prevention Science*, *4*, 55–63. https://doi.org/10.1023/A:1021786811186	(Kam et al. 2003)	360	0
7	Denham, S. A., & Brown, C. (2010). “Plays nice with others”: Social–emotional learning and academic success. *Early Education and Development*, *21*(5), 652–680. https://doi.org/10.1080/10409289.2010.497450	(Denham and Brown 2010)	332	19
8	Jagers, R. J., Rivas-Drake, D., & Williams, B. (2019). Transformative social and emotional learning (SEL): Toward SEL in service of educational equity and excellence. *Educational Psychologist*, *54*(3), 162–184. https://doi.org/10.1080/00461520.2019.1623032	(Jagers et al. 2019)	327	0
9	Schonert-Reichl, K. A. (2017). Social and emotional learning and teachers. *The Future of Children*, 137–155. https://doi.org/10.1353/foc.2017.0007	(Schonert-Reichl 2017)	312	15
10	Payton, J. W., Wardlaw, D. M., Graczyk, P. A., Bloodworth, M. R., Tompsett, C. J., & Weissberg, R. P. (2000). Social and emotional learning: A framework for promoting mental health and reducing risk behavior in children and youth. *Journal of School Health*, *70*(5), 179–185. https://doi.org/10.1111/j.1746-1561.2000.tb06468.x	(Payton et al. 2000)	306	0

## Data Availability

The data presented in this study are available on request from the author.

## Supplementary Materials

The following supporting information can be downloaded at https://www.mdpi.com/article/10.3390/jintelligence13090123/s1, Table S1: Unified top 50 keywords; Table S2: Frequently used keywords within the densest thematic clusters; Table S3: Thematic evolution of SEL research in primary education (1983–2014 vs. 2015–2025).
