# Supplementary material for: Mapping the Evolution of Social and Emotional Learning Research in Primary Education Contexts: A Bibliometric and Thematic Analysis
Source: J Intell. 2025 Sep 19;13(9):123. doi: 10.3390/jintelligence13090123 (PMC12471157; doi:10.3390/jintelligence13090123)
Supplement: Supplementary file 1 [file jintelligence-13-00123-s001.zip › jintelligence-3801147-supplementary.pdf]

Supplementary Materials: Mapping the Evolution of Social and Emotional Learning Research in Primary Education Contexts: A Bibliometric and Thematic Analysis

Melek Alemdar[1,2]

[1] Ahmet Keleşoğlu Education Faculty, Necmettin Erbakan University, 42090 Meram/Konya, Türkiye; melek.alemdar@erbakan.edu.tr
[2] Manchester Institute of Education, The University of Manchester, M13 9PL, Manchester, UK; melek.alemdar@manchester.ac.uk

Table S1. Unified Top 50 Keywords.

Unified Term	Included Variants	Total Frequency
Social and Emotional Learning (SEL)	social and emotional learning (287), social-emotional learning (206), social emotional learning (112), socio-emotional learning (32), social–emotional learning (15), social-emotional (21), emotional learning (19), social and emotional (13), SEL (39)	744
Early Childhood / Preschool	preschool (43), early childhood (38), early childhood education (27)	108
Adolescence / Adolescents	adolescents (33), adolescence (23)	56
Education and schooling	education (46), learning (32), pedagogy (14), curriculum (14), school (27), schools (25), elementary school (28), primary school (21)	207
Mental Health & wellbeing	mental health (61), wellbeing (18), well-being (16), resilience (22), mindfulness (50)	167
Children	children (57), child development (15)	72
Intervention/ Implementation	prevention (35), intervention (49), implementation (34), program evaluation (14), assessment (13)	145
Emotional Intelligence	emotional intelligence (27), emotion regulation (15), self-regulation (25)	67
COVID-19	covid-19 (24)	24
Social Skills	social skills (20), social-emotional competence (17), social (19), empathy (16)	72
Academic Achievement	academic achievement (17)	17
Autism	autism (17)	17
Teachers & Professional development	teachers (16), professional development (13)	29
Bullying	bullying (14)	14
Aggression	aggression (13)	13

Note. This table presents the unified top 50 most frequent keywords identified across the dataset. Variants were grouped under common conceptual categories.

Table S2. Frequently Used Keywords Within the Densest Thematic Clusters.

	Themes	Clusters	Terms
1	Motor	Social skills	Social skills, curriculum, life skills, social-emotional development, emotional, emotion knowledge
2	Nich	Professional development	Professional development, implementation science, literacy, early childhood education and care
3	Emerging or Declining	Preschool children	Preschool children, school readiness
4	Basic	Social emotional learning	Social emotional learning, mental health, children, mindfulness, intervention, sel, prevention, implementation, adolescents, emotional intelligence, schools, covid-19, adolescence, resilience, primary school, social, wellbeing, social-emotional competence, well-being, emotion regulation, pedagogy, aggression, assessment, universal intervention, evaluation, interventions, social-emotional skills, school-based intervention, middle school, parenting, teacher, elementary, measurement, trauma, emotional literacy, school-based, students, universal prevention

Table S3. Thematic evolution of SEL research in primary education (1983–2014 vs. 2015–2025).

**Initial Theme (1983–2014)**	**Later Theme (2015–2025)**	**Evolution Type**	**Notes/Interpretation**	**Occurrences**	**Weighted Inclusion Index**	**Stability Index**
Social and emotional learning	Social and emotional learning	Stable, central	Remained dominant, overarching theme	42	0.89	0.01
Early intervention	Early intervention	Stable	Consistently emphasised across both periods	2	1.00	0.11
Teacher	Professional development	Evolved	Shift from teacher role to teacher training/professionalisation	2	1.00	0.25
Social competence	Child development	Evolved	Focus broadened to developmental outcomes	3	0.33	0.07
Adolescents	Physical education	Shifted linkage	Adolescence research tied to PE contexts	2	1.00	0.25
—	Gender	Emerging	New standalone theme in recent years	4	0.07	0.05
—	Preschool	Emerging	Greater focus on early years/pre-primary	5	0.20	0.02
Self-regulation	Social and emotional learning	Absorbed	Integrated into the broader SEL theme	2	1.00	0.02
Assessment	Social and emotional learning	Absorbed	Folded into general SEL discourse	2	0.75	0.02
Pedagogy / Responsive classroom approach	Social and emotional learning	Absorbed	Incorporated under the umbrella of SEL	3	0.53	0.02
